# Evaluation of gilteritinib in combination with chemotherapy in preclinical models of *FLT3-ITD^+^* acute myeloid leukemia

**DOI:** 10.18632/oncotarget.26811

**Published:** 2019-04-02

**Authors:** Yoko Ueno, Masamichi Mori, Yoshiteru Kamiyama, Rika Saito, Naoki Kaneko, Eriko Isshiki, Sadao Kuromitsu, Masahiro Takeuchi

**Affiliations:** ^1^ Drug Discovery Research, Astellas Pharma, Inc., Ibaraki, Japan; ^2^ Biological Research Division, Astellas Research Technologies Co., Ltd., Ibaraki, Japan

**Keywords:** acute myeloid leukemia, FLT3 inhibition, internal tandem duplication, combination therapy, apoptosis

## Abstract

Activating *internal tandem duplication (ITD)* and *tyrosine kinase domain (TKD)* point mutations in *Fms-like tyrosine kinase 3* (*FLT3*) occur in approximately 30% of patients with acute myeloid leukemia (AML), and confer a poor prognosis with standard cytarabine/anthracycline or azacitidine-based chemotherapy regimens. Gilteritinib is a highly-specific, potent FLT3/AXL inhibitor with demonstrated activity against *FLT3-ITD* and *FLT3-TKD* mutations. Compared with salvage chemotherapy, treatment with once-daily oral gilteritinib demonstrated a clinical benefit in patients with *FLT3*-mutated relapsed/refractory AML, which led to its recent approval in Japan and the United States. We investigated the effects of gilteritinib combined with cytarabine plus daunorubicin/idarubicin, or combined with azacitidine in human *FLT3-ITD*–positive (*FLT3-ITD*^+^) AML cell lines and xenografted mouse models. Gilteritinib induced G_1_ arrest and apoptosis in a dose-dependent manner. The addition of cytarabine, daunorubicin, idarubicin, or azacitidine potentiated apoptosis. Gilteritinib alone or combined with cytarabine, daunorubicin, idarubicin, or azacitidine, inhibited anti-apoptotic protein expression in MV4-11 cells. In xenografted mice, administration of cytarabine, idarubicin, or azacitidine in combination with gilteritinib had little impact on plasma or intratumor PK profiles of gilteritinib, cytarabine, idarubicin, or azacitidine. Gilteritinib combined with chemotherapy reduced tumor volume to a greater extent than either gilteritinib or chemotherapy alone. Of note, the addition of cytarabine plus daunorubicin/idarubicin led to tumor regression in mice, with complete regression observed in six out of eight mice in both triple combination groups. These findings support the investigation of gilteritinib combined with chemotherapy in patients with *FLT3-ITD*^+^ AML, including those who are ineligible for intensive chemotherapy.

## INTRODUCTION

Acute myeloid leukemia (AML) is characterized by the clonal expansion of immature myeloid cells as a consequence of a multistep transformation of hematopoietic precursor cells that allows them to progress through endless cell cycles and become resistant to cell death [1]. The hyperproliferation of immature myeloid cells increases the risk of genetic damage by compromising DNA damage response pathways and weakening checkpoints, giving way to further genetic anomalies that lead to disease progression [1]. Standard chemotherapy regimens are not effective in all patients with newly diagnosed AML, and additional targeted therapies are often required.

An inadequate response to standard chemotherapy regimens often stems from the overexpression of *Fms*-like tyrosine kinase 3 (FLT3) receptors in patients with AML [2]. Activating mutations in *FLT3*, such as internal tandem duplications (ITDs) in the juxtamembrane domain of the FLT3 receptor and tyrosine kinase domain (TKD) mutations, occur in approximately 30% of adults and elderly patients with AML and induce ligand-independent proliferation stemming from constitutive activation of the FLT3 receptor [3–5]. The presence of *FLT3-ITD* mutations is associated with high rates of relapse and poor overall survival after standard intensive cytarabine (AraC)/anthracycline chemotherapy [6–8]. Treatment resistance in patients with *FLT3* mutation-positive AML is also facilitated by the overexpression of the FLT3 ligand, anti-apoptotic proteins, and AXL, an oncogenic tyrosine kinase that facilitates FLT3 activation [9–12].

FLT3 inhibitors have been shown to induce cytotoxic effects and suppress the growth of primary leukemic blasts from patients with *FLT3-ITD*^+^ AML and BA/F3 cells expressing constitutively active FLT3 [13–15]. Evidence suggests that the epigenetic modulator, azacitidine (Aza), which is administered as an alternative to standard intensive chemotherapy in elderly patients with AML [16], does not upregulate FLT3 ligand and may in fact enhance the efficacy of FLT3 inhibitors [17, 18]. Gilteritinib (ASP2215) is a novel oral small molecule FLT3/AXL inhibitor [19]. *In vitro*, gilteritinib demonstrated highly specific potent inhibition of FLT3 receptors with activating ITD mutations [19, 20]. In preclinical models of *FLT3*-mutated AML, gilteritinib demonstrated potent antitumor effects when administered as a single agent [19]. In a phase 1/2 study, once-daily gilteritinib administered as a single agent resulted in high response rates and improved overall survival in *FLT3* mutation-positive patients with relapsed/refractory (R/R) AML [21]. Gilteritinib was recently approved in Japan and the United States based on findings from a phase 3 study (NCT02421939), which demonstrated the clinical benefit of gilteritinib compared with salvage chemotherapy in patients with *FLT3* mutation-positive R/R AML [22, 23].

To determine whether combining gilteritinib with chemotherapy potentiates the cytotoxic effects of chemotherapy, which may help to overcome treatment resistance, we examined the effects of gilteritinib in combination with AraC plus daunorubicin (DNR) or idarubicin (IDR), or in combination with Aza in preclinical models of AML. Cell cycle and apoptotic effects were investigated in an AML cell line that exclusively expressed the *FLT3-ITD* allele and in one that expressed both mutated and wild-type *FLT3*; the PK and antitumor effects were examined in a *FLT3-ITD*^+^ AML mouse model.

## RESULTS

### Gilteritinib induces cell cycle effects and apoptosis

**Figure 1:**
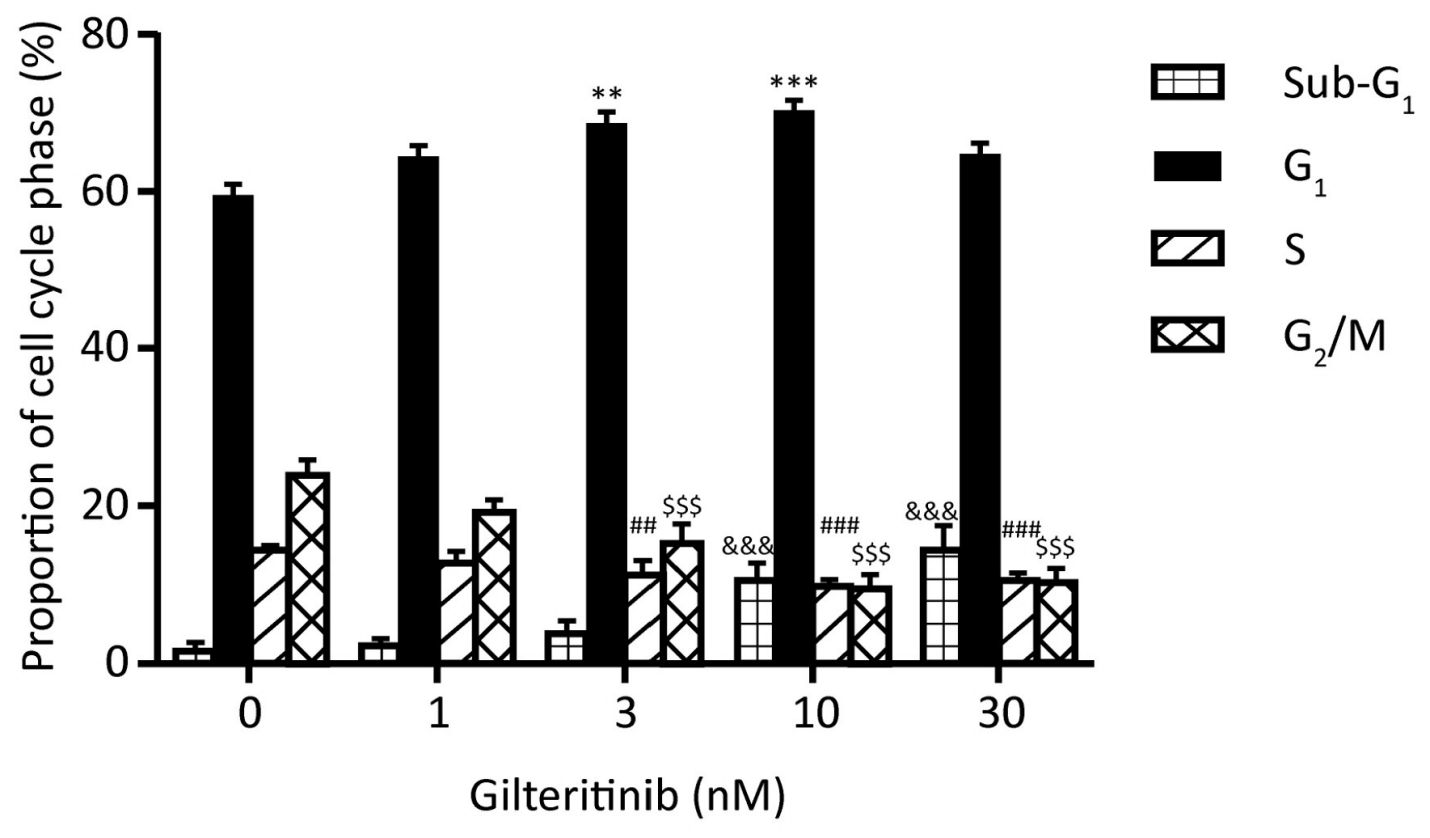
Cell cycle effects of gilteritinib in MV4-11 cells MV4-11 cells were treated with vehicle (DMSO) or with gilteritinib (1, 3, 10, and 30 nM) for 24 hours. The percentages of cells in sub-G1, G1, S, and G2/M phases following gilteritinib treatment were compared with those in the vehicle group using Dunnett's multiple comparison test using within subject error. Vertical bars represent mean values ± SEM from four independent assays. ^**^P<0.01 versus the percentage of cells in the G1 phase in the vehicle group. ^***^P<0.001 versus the percentage of cells in the G1 phase in the vehicle group. ^&&&^*P*<0.001 compared with the percentage of cells in the sub-G1 phase in the vehicle group. ^##^*P*<0.01 and ^###^*P*<0.001 compared with the percentage of cells in the S phase in the vehicle group. ^$$$^*P*<0.001 compared with the percentage of cells in the G2/M phase in the vehicle group.

We first evaluated the effects of gilteritinib on the cell cycle. The mean proportion of MV4-11 cells in G1 phase were significantly increased at gilteritinib concentrations of 3 (69.0%; *P*<0.01) and 10 nM (70.7%; *P*<0.001) relative to the proportion of MV4-11 cells in the G1 phase observed with control treatment (59.8%) (Figure 1).

**Figure 2:**
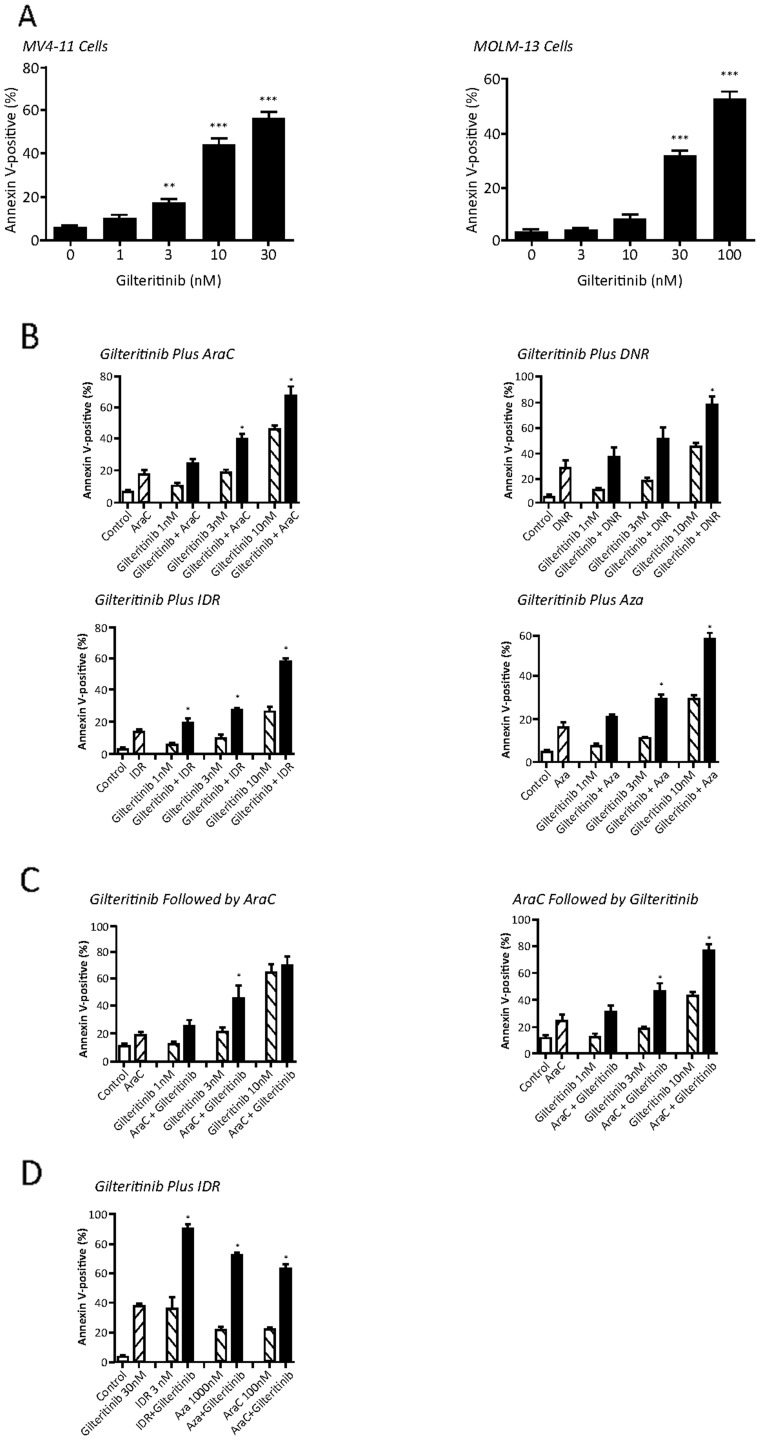
Induction of apoptosis by gilteritinib in MV4-11 cells and MOLM-13 cells **(A)** MV4-11 cells were treated with gilteritinib (1, 3, 10, and 30 nM) for 48 hours and MOLM-13 cells were treated with gilteritinib (1, 3, 10, 30, and 100 nM) for 48 hours. The percentage of annexin-V-positive stained cells was used as an index of apoptosis. For MV4-11 cell experiments (left), each bar represents the mean value ± SEM from four independent assays. For MOLM-13 cell experiments (right), each bar represents the mean value ± SEM from three independent assays. ^**^P<0.01 and ^***^P<0.001 versus the vehicle-treated group (0 nM). **(B)** MV4-11 cells were treated with gilteritinib (1, 3, and 10 nM) either alone or in combination with either AraC (1000 nM), DNR (6 nM), IDR (1 nM), or Aza (1000 nM); AraC (1000 nM), DNR (6 nM), IDR (1 nM), or Aza (1000 nM) alone; or vehicle (control; DMSO) for 48 hours. Vertical bars represent the mean values ± SEM from three independent assays. ^*^Combination treatment was significantly more potent than treatment with the single agent alone. **(C)** MV4-11 cells were sequentially treated with gilteritinib (1, 3, and 10 nM) followed by AraC (1000 nM) (left) or sequentially treated with AraC (1000 nM) followed by gilteritinib (1, 3, and 10 nM) (right) for 48 hours. Each bar represents the mean value ± SEM from three independent assays. ^*^Combination treatment was significantly more potent than treatment with the single agent alone. **(D)** MOLM-13 cells were treated with gilteritinib (30 nM), IDR (3 nM), AraC (100 nM), Aza (1000 nM), or vehicle (DMSO). Each bar represents the mean value ± SEM from three independent assays. ^*^Combination treatment was significantly more potent than treatment with the single agent alone. Abbreviations: AraC, cytarabine; Aza, azacitidine; DNR, daunorubicin; IDR, idarubicin.

We then examined the cytotoxic effects of gilteritinib by evaluating the induction of apoptosis in MV4-11 cells. The proportion of annexin-V-positive cells significantly increased from baseline, reflecting the induction of apoptosis in MV4-11 cells expressing *FLT3-ITD* mutations following 48 hours of treatment with gilteritinib at concentrations of 3 nM (*P*<0.01 vs vehicle) 10 nM (*P*<0.001 vs vehicle) and 30 nM (*P*<0.001 vs vehicle) (Figure 2A). In MV4-11 cells treated with gilteritinib plus AraC (1000 nM) or gilteritinib plus Aza (1000 nM), a significant increase in annexin-V-positive cells was observed at gilteritinib concentrations of 3 nM (AraC combination: 39.8%; Aza combination: 29.6%) and 10 nM (AraC combination: 67.5%; Aza combination: 57.2%) (Figure 2B). A significant increase in the mean percentage of annexin-V-positive cells relative to control (6.9%) was observed with 10 nM gilteritinib in combination with 6 nM DNR (78.6%). The mean percentage of annexin-V-positive cells was significantly increased in cells treated with gilteritinib in combination with 1 nM IDR at gilteritinib concentrations of 1 (21.2%), 3 (28.8%), and 10 nM (59.8%) compared with each single agent (Figure 2B). To determine whether pretreatment with gilteritinib inhibited the cytotoxic effects of AraC, we investigated the effect of sequential treatment on apoptosis. Sequential treatment of MV4-11 cells with gilteritinib followed by AraC or with AraC followed by gilteritinib significantly increased the mean percentage (±SEM) of annexin-V-positive cells at 3 nM concentrations of gilteritinib when compared with AraC or gilteritinib alone (Figure 2C). In MOLM-13 cells, treatment with gilteritinib alone resulted in significant increases in the percentage of annexin V-positive cells at concentrations of 30 nM (32.0%) and 100 nM (52.4%) versus control (4.1%) (*P*<0.001; Figure 2A). Treatment with gilteritinib (30 nM) plus IDR (3 nM), AraC (100 nM), or Aza (1000 nM) resulted in significant increases in the mean percentages of annexin V-positive cells (91.4%, 65.1%, and 73.5%, for gilteritinib plus IDR, AraC, and Aza, respectively) compared with treatment with IDR, AraC, or Aza alone (36.6%, 21.9%, 21.9%, for IDR, AraC, and and Aza, respectively; Figure 2D).

### Gilteritinib inhibits the expression of anti-apoptotic proteins

**Figure 3:**
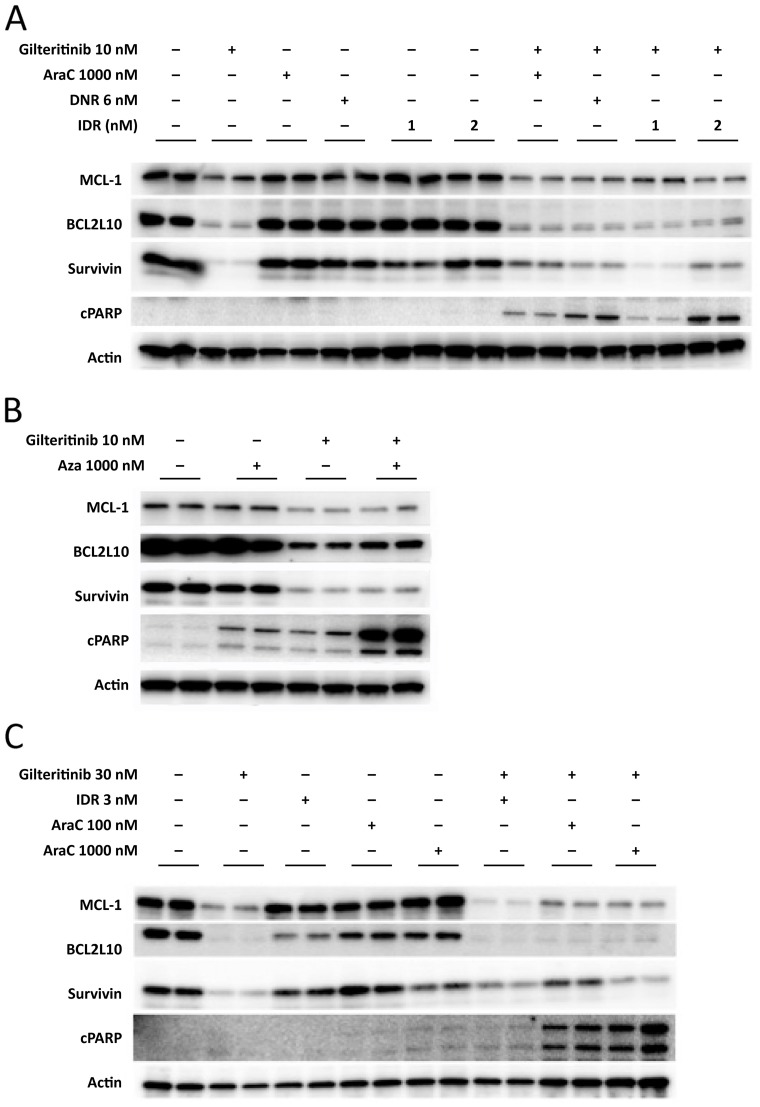
Inhibition of anti-apoptotic protein expression in MV4-11 cells and MOLM-13 cells **(A)** Western blot analysis of anti-apoptotic protein expression in MV4-11 cells after 24-hour treatment with gilteritinib (10 nM), AraC (1000 nM), DNR (6 nM), or IDR (1 nM and 2 nM) alone, or as combination treatment, is depicted. **(B)** Western blot analysis of anti-apoptotic protein expression in MV4-11 cells after 24-hour treatment with gilteritinib (10 nM) or Aza (1000 nM) alone, or as combination treatment, is depicted. **(C)** Western blot analysis of anti-apoptotic protein expression in MOLM-13 cells after 24-hour treatment with gilteritinib (30 nM), AraC (100 nM), IDR (3 nM), or Aza (1000 nM) alone, or gilteritinib in combination with these agents. Abbreviations: AraC, cytarabine; Aza, azacitidine; cPARP, cleaved poly-ADP ribose polymerase; DNR, daunorubicin hydrochloride; IDR, idarubicin hydrochloride.

To better understand the mechanism of enhanced sensitivity of tumor cells to gilteritinib when administered in combination with chemotherapy, we evaluated anti-apoptotic protein expression in MV4-11 cells. Treatment with gilteritinib (10 nM) alone inhibited the expression of MCL-1, BCL2L10, and survivin (Figure 3A), whereas treatment with chemotherapy alone had no apparent effect on anti-apoptotic protein expression. Combination treatment with 10 nM gilteritinib plus 1000 nM AraC, or with gilteritinib plus DNR (6 nM) or IDR (1 nM and 2 nM) also inhibited the expression of MCL-1, BCL2L10, and survivin and induced the upregulation of cPARP expression, a marker of apoptosis (Figure 3A). In MV4-11 cells treated with Aza (1000 nM) alone, there was no change in anti-apoptotic protein expression, whereas gilteritinib (10 nM) or gilteritinib (10 nM) plus Aza (1000 nM), decreased expression of MCL-1, BCL2L10, and survivin (Figure 3B). Expression of cPARP was increased in cells treated with gilteritinib plus Aza (Figure 3B). Western blot analyses of MOLM-13 cell lysates (Figure 3C) showed that treatment with gilteritinib alone strongly inhibited the expression of MCL-1, BCL2L10, and survivin. Treatment with AraC, IDR or Aza had little effect on BCL2L10, MCL-1 or survivin expression. Combination treatment with gilteritinib plus AraC, IDR, or Aza inhibited expression of MCL-1, BCL2L10, and survivin, and upregulated the expression of cPARP.

### Pharmacokinetic profiles of gilteritinib in xenografted nude mice

**Figure 4:**
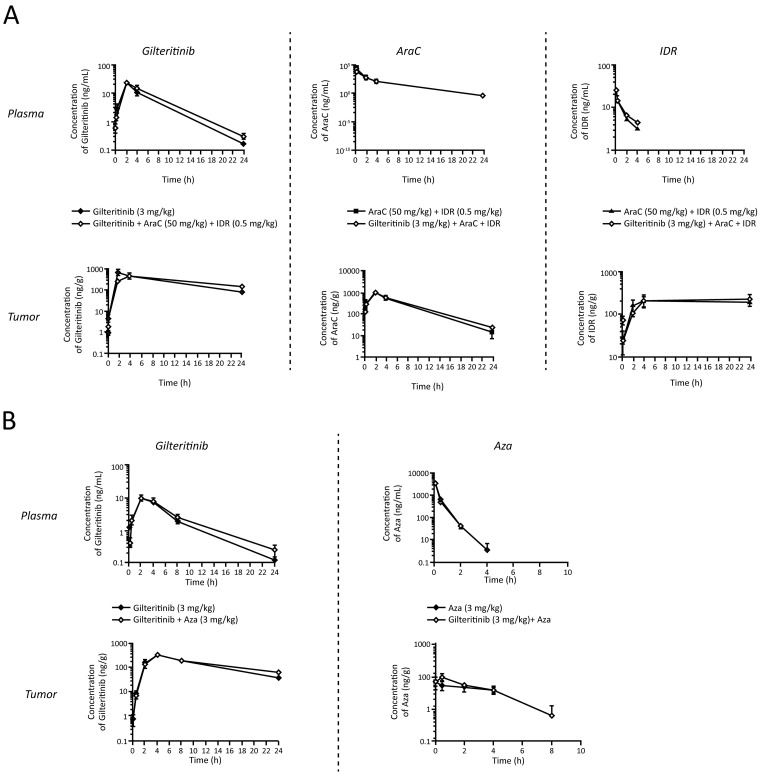
Pharmacokinetic profiles of gilteritinib, AraC, IDR, and Aza in xenografted nude mice **(A)** The plasma and intratumor concentrations of gilteritinib, AraC, and IDR in xenografted mice are shown. **(B)** The plasma and intratumor concentrations of gilteritinib and Aza in xenografted mice are displayed. Data are expressed as mean ± SEM using n=3 per time point for all post-dose time points except for the plasma fraction of the gilteritinib plus Aza combination 5-minute post-dose time point, which was based on n=2. Abbreviations: AraC, cytarabine; Aza, azacitidine; IDR, idarubicin; SEM, standard error of the mean.

There was no discernable difference in the PK profile of gilteritinib when administered alone or in combination with AraC/IDR or Aza. Plasma concentrations of gilteritinib peaked at 2 hours after administration and declined over a 24-hour period thereafter regardless of whether the drug was administered alone or in combination with AraC/IDR (Figure 4A), or with Aza (Figure 4B). Intratumor concentrations of gilteritinib also peaked between 2 and 4 hours after administration and declined only slightly over a 24-hour period in mice receiving gilteritinib alone, triple combination therapy, or gilteritinib plus Aza (Figure 4A and 4B). Plasma concentrations of AraC declined gradually from the time of administration over a 24-hour period in both AraC plus IDR and triple combination therapy groups (Figure 4A). Specific plasma and intratumor PK parameters following triple combination treatment and treatment with gilteritinib plus Aza in xenografted mice are presented in Tables 1, 2, and 3. Concentrations of gilteritinib and IDR were higher in the tumor fraction than in the plasma fraction whereas the concentration of AraC was higher in the plasma fraction. Across all combination treatments, gilteritinib concentrations were higher and persisted over a longer duration in the tumor fraction than in the plasma fraction.

**Table 1:** Pharmacokinetic parameters of gilteritinib and AraC in nude mice xenografted with MV4-11 cells after combination treatment

Gilteritinib							
**Fraction**	**Dose (mg/kg)**			**PK Parameter**			
**Plasma**	**Gilteritinib**	**AraC**	**IDR**	**T_max_** **(h)**	**C_max_** **(ng/mL)**	**AUC_24_** **(ng·h/mL)**	**AUC_24_ ratio**
	3	0	0	2	21.33	156.3	1.28
	3	50	0.5	2	22.27	199.4	
**Tumor**	**Gilteritinib**	**AraC**	**IDR**	**T_max_** **(h)**	**C_max_** **(ng/g)**	**AUC_24_** **(ng·h/g)**	**AUC_24_ ratio**
	3	0	0	2	677.0	6430.2	1.03
	3	50	0.5	4	436.7	6639.0	
**AraC**							
**Fraction**	**Dose (mg/kg)**			**PK Parameter**			
**Plasma**	**Gilteritinib**	**AraC**	**IDR**	**T_max_** **(h)**	**C_max_** **(ng/mL)**	**AUC_24_** **(ng·h/mL)**	**AUC_24_ ratio**
	0	50	0.5	0.25	28730	31598.4	0.32
	3	50	0.5	0.083	17970	9959.5	
**Tumor**	**Gilteritinib**	**AraC**	**IDR**	**T_max_** **(h)**	**C_max_** **(ng/g)**	**AUC_24_** **(ng·h/g)**	**AUC_24_ ratio**
	0	50	0.5	2	904.3	7968	1.13
	3	50	0.5	2	1055	899	

Abbreviations: AraC, cytarabine; AUC_24_, area under the time-concentration curve from time 0 to 24 hours; C_max_, maximum concentration; IDR, idarubicin; PK, pharmacokinetic; T_max_, time to reach maximum concentration.

**Table 2:** Pharmacokinetic parameters of idarubicin in nude mice xenografted with MV4-11 cells after combination treatment

Fraction	Dose (mg/kg)			PK Parameter				
	**Gilteritinib**	**AraC**	**IDR**	**T_1/2_** **(h)**	**CL_tot_** **(L/h/kg)**	**V_ss_** **(L/kg)**	**AUC_24_** **(ng·h/mL)**	**AUC_24_** **Ratio**
**Plasma**	0	50	0.5	1.49	13.03	26.7	63.26	1.23
	3	50	0.5	2.24	10.34	31.5	77.92	
	**Gilteritinib**	**AraC**	**IDR**	**T_max_** **(h)**	**C_max_** **(ng/g)**	**C_max_ ratio**	**AUC_24_** **(ng·h/g)**	**AUC_24_** **Ratio**
**Tumor**	0	50	0.5	4	203.7	1.11	4491	1.08
	3	50	0.5	24	225.7		4855	

Abbreviations: AraC, cytarabine; AUC_24_, area under the time-concentration curve from time 0 to 24 hours; CL_tot_, total clearance; C_max_, maximum concentration; IDR, idarubicin; PK, pharmacokinetic; T_1/2_, half-life; V_ss_, volume of drug distribution at steady state.

**Table 3:** Pharmacokinetic parameters of gilteritinib and azacitidine in nude mice xenografted with MV4-11 cells after combination treatment

Gilteritinib						
**Fraction**	**Dose (mg/kg)**		**PK Parameter**			
**Plasma**	**Gilteritinib**	**Azacitidine**	**T_max_** **(h)**	**C_max_** **(ng/mL)**	**AUC_24_** **(ng·h/mL)**	**AUC_24_ ratio**
	3	0	2	11.0	65.8	1.05
	3	3	2	10.1	69.1	
**Tumor**	**Gilteritinib**	**Azacitidine**	**T_max_** **(h)**	**C_max_** **(ng/g)**	**AUC_24_** **(ng·h/g)**	**AUC_24_ ratio**
	3	0	4	387	3821	1.04
	3	3	4	349	3964	
**Azacitidine**						
**Fraction**	**Dose (mg/kg)**		**PK Parameter**			
**Plasma**	**Gilteritinib**	**Azacitidine**	**T_max_** **(h)**	**C_max_** **(ng/mL)**	**AUC_24_** **(ng·h/mL)**	**AUC_24_ ratio**
	3	0	0.08	3390	1849	0.87
	3	3	0.08	3440	1614	
**Tumor**	**Gilteritinib**	**Azacitidine**	**T_max_** **(h)**	**C_max_** **(ng/g)**	**AUC_24_** **(ng·h/g)**	**AUC_24_ ratio**
	3	0	0.08	61.3	323	1.17
	3	3	0.5	96.8	378	

Abbreviations: AUC_24_, area under the time-concentration curve from time 0 to 24 hours; C_max_, maximum concentration; PK, pharmacokinetic; T_max_, time to reach maximum concentration.

### Antitumor effects of gilteritinib in combination with chemotherapy

**Figure 5:**
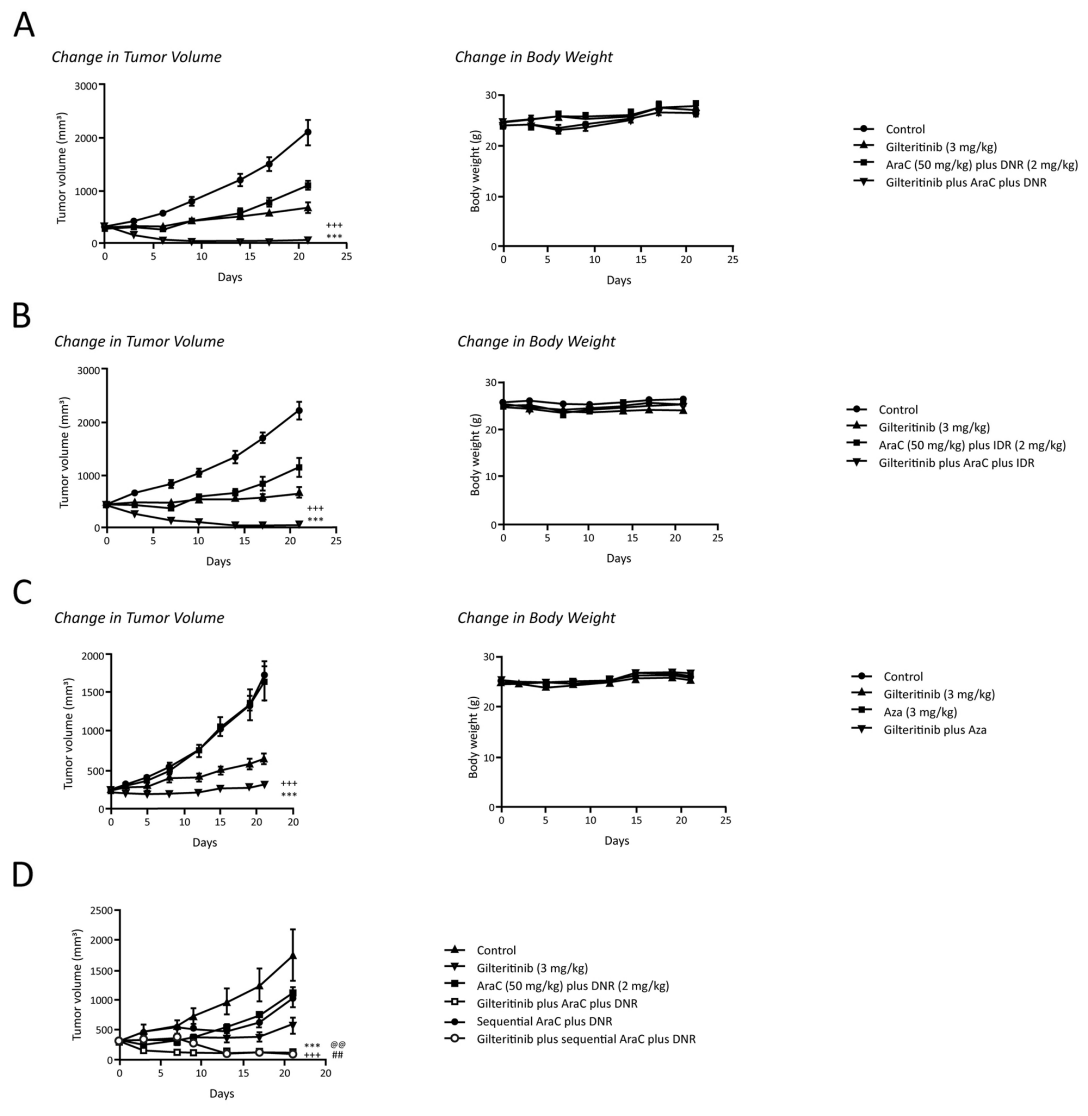
Antitumor effects of gilteritinib in combination with chemotherapy in mice xenografted with MV4-11 cells **(A)** Xenografted mice were treated with once-daily oral gilteritinib (3 mg/kg/day) on Days 0 to 20, intraperitoneal AraC (50 mg/kg/day) on Days 0 to 4, and intravenous DNR (2 mg/kg/day) on Days 0 to 2. Data are presented as mean ± SEM using n=8 per time point. ^***^P<0.001 compared with treatment with gilteritinib alone; ^+++^*P*<0.001 compared with treatment with AraC plus DNR only. **(B)** Xenografted mice were treated with once-daily oral gilteritinib (3 mg/kg/day, Days 0–20), IP AraC (50 mg/kg/day, Days 0–4), and IV IDR (0.5 mg/kg/day, Days 0–2). Data are presented as mean ± SEM using n=8 per time point. ^***^P<0.001 compared with treatment with gilteritinib alone; ^+++^*P*<0.001 compared with treatment with AraC plus IDR only. **(C)** Xenografted mice were treated with once-daily oral gilteritinib (3 mg/kg/day, Days 0–20), and IV Aza (3 mg/kg/day, Days 0–4). Data are presented as mean ± SEM using n=10 per time point. ^***^P<0.001 compared with treatment with gilteritinib alone; ^+++^*P*<0.001 compared with treatment with Aza alone. **(D)** Xenografted mice were treated orally with gilteritinib (3 mg/kg/day, Days 0–20) IP AraC (50 mg/kg/day, Days 0–4) plus IV DNR (2 mg/kg/day, Days 0–2), sequential administration of gilteritinib (3 mg/kg/day, Days 0–20) followed by AraC (50 mg/kg/day, Days 7 –11) plus DNR (2 mg/kg/day, Days 7–9); ^***^P<0.001 for triple combination therapy group versus AraC plus DNR only; ^+++^*P*<0.001 for sequential triple combination therapy versus sequential AraC plus DNR only; ^@@^*P*<0.01 triple combination group versus gilteritinib alone; ^##^*P*<0.01: sequential triple combination group versus gilteritinib alone. Abbreviations: AraC, cytarabine; Aza, azacitidine; DNR, daunorubicin; IDR, idarubicin.

We investigated the effects of gilteritinib in combination with AraC/DNR, AraC/IDR, and Aza in mice xenografted with MV4-11 cells or MOLM-13 cells. At Day 21, 78% growth inhibition of MV4-11 tumors was observed with gilteritinib alone; 56% growth inhibition was observed with AraC plus DNR (Table 4). Triple combination therapy with gilteritinib plus AraC and DNR completely inhibited tumor growth and induced tumor regression by 80%, with complete remission in six of eight mice (Table 4). Tumor volume on Day 21 in the triple combination therapy group was significantly smaller than that in either the gilteritinib or AraC plus DNR treatment groups (Figure 5A). Mean (±SEM) tumor weights on Day 21 were 1.234±0.150 g, 0.441±0.076 g, 0.693±0.065 g, and 0.018±0.012 g, in control, gilteritinib, AraC plus DNR, and triple combination therapy groups, respectively. Furthermore, sequential administration of gilteritinib followed by AraC plus DNR, or gilteritinib followed by AraC and subsequently DNR, also led to significant reductions in tumor volume compared with gilteritinib alone and AraC plus DNR only (Figure 5D).

**Table 4:** Inhibition of tumor growth and tumor regression on day 21 following treatment with gilteritinib alone and combination treatment in mice xenografted with MV4-11 or MOLM-13 cells

Treatment Group	Inhibition of Tumor Growth (%)	Regression of Tumor (%)	Mice with Complete Tumor Regression, n
**Mice Xenografted With MV4-11 Cells**			
**Gilteritinib plus AraC and DNR**			
Gilteritinib	78	0	0
AraC plus DNR	56	0	0
Gilteritinib plus AraC and DNR	>100	80	6 of 8
**Gilteritinib plus AraC and IDR**			
Gilteritinib	85	0	0
AraC plus IDR	60	0	0
Gilteritinib plus AraC and IDR	>100	92	6 of 8
**Gilteritinib plus Aza**			
Gilteritinib	71	0	0
Aza	5	0	0
Gilteritinib plus Aza	94	0	0
**Mice Xenografted With MOLM13 Cells**			
**Gilteritinib plus AraC and IDR**			
Gilteritinib	93	-	0
IDR plus AraC	12	-	0
Gilteritinib plus AraC plus IDR	-	50	0
**Gilteritinib plus Aza**			
Gilteritinib	90	-	0
Aza	37	-	0
Gilteritinib plus Aza	-	46	0

Abbreviations: AraC, cytarabine; Aza, azacitidine; DNR, daunorubicin; IDR, idarubicin.

**Figure 6:**
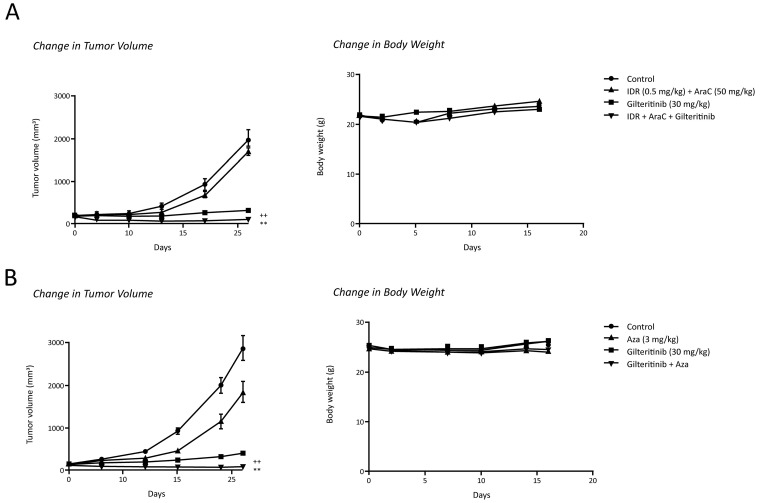
Antitumor effects of gilteritinib in combination with chemotherapy in mice xenografted with MOLM-13 cells **(A)** Xenografted mice were treated with once-daily oral gilteritinib (30 mg/kg/day) on Days 0 to 15, IP AraC (50 mg/kg/day) on Days 0 to 4, and IV IDR (0.5 mg/kg/day, Days 0–2). Body weight was assessed on Days 0, 2, 5, 8, 12, and 16. Data are presented as mean ± SEM using n=10 per time point. ^++^*P*<0.001 versus combination treatment group; ^**^P<0.001 versus gilteritinib alone. **(B)** Xenografted mice were treated with once-daily oral gilteritinib (30 mg/kg/day) on Days 0 to 16 and IV Aza (3 mg/kg/day, Days 0–4). Body weight was assessed on Days 0, 2, 7, 10, 14, and 16. Data are presented as mean ± SEM using n=10 per time point. ^++^*P*<0.001 versus Aza alone; ^**^P<0.001 versus gilteritinib alone. Abbreviations: AraC, cytarabine; Aza, azacitidine; IDR, idarubicin.

Treatment with gilteritinib alone and treatment with AraC plus IDR inhibited tumor growth by 85% and 60%, respectively, by Day 21 (Table 4). Triple combination therapy with gilteritinib plus AraC and IDR completely inhibited tumor growth and induced tumor regression by 92%; complete regression was observed in six of eight mice (Table 4). Tumor volume on Day 21 in the triple combination therapy group was significantly smaller than that in the gilteritinib and AraC plus IDR treatment groups (Figure 5B). Mean tumor weights on Day 21 were 1.64±0.12 g, 0.53±0.09 g, 0.77±0.13 g, and 0.02±0.01 g in control, gilteritinib, AraC plus IDR, and triple combination therapy groups, respectively. At Day 21, treatment with gilteritinib, Aza, or both agents inhibited tumor growth by 71%, 5%, and 94%, respectively (Table 4). Tumor volume on Day 21 in the gilteritinib plus Aza group was significantly smaller than that in either the gilteritinib or Aza treatment groups (Figure 5C). No significant differences in body weight were observed on Day 21 between the control, gilteritinib, AraC plus DNR, AraC plus IDR, or triple combination therapy groups, or between control, gilteritinib, Aza, and gilteritinib plus Aza treatment groups. Treatment with gilteritinib (30 mg/kg) alone inhibited tumor growth by ≥90% by Day 16 in mice xenografted with MOLM-13 cells (Table 4). Treatment with AraC plus IDR or Aza alone resulted in 12% and 37% inhibition of tumor growth, respectively. Combination therapy with gilteritinib plus AraC and IDR or gilteritinib plus Aza resulted in >45% tumor regression (Table 4, Figure 6A and 6B). There was little change in body weight over time in any treatment group (Figure 6A and 6B).

## DISCUSSION

Patients with *FLT3* mutation-positive AML present a clinical challenge, given the diminished likelihood of a durable response and long-term survival with standard chemotherapy and high relapse rates. Therapies that specifically target FLT3 are therefore essential to improve clinical outcomes and survival in this vulnerable patient population. Gilteritinib has demonstrated robust FLT3 inhibition in human AML cell lines and induced strong antileukemic responses in *FLT3-ITD*^+^ patients with relapsed/refractory AML when administered as a single agent [21]. Given that some patients may not adequately respond to single-agent gilteritinib but may benefit from gilteritinib plus chemotherapy, we evaluated the feasibility of combining gilteritinib with AraC/anthracycline or with Aza in preclinical cellular and animal models of *FLT3-ITD*^+^ AML.

Findings from this preclinical study showed that treatment with gilteritinib resulted in an increase in the proportion of *FLT3-ITD*^+^ MV4-11 cells in the G_1_ phase, induction of apoptosis and the downregulation of anti-apoptotic protein expression. Combining gilteritinib with AraC, DNR, IDR, or Aza potentiated apoptosis in MV4-11 cells. Similar effects were noted in *FLT3-ITD*^+^ MOLM-13 cells treated with gilteritinib alone or in combination with AraC, IDR, or Aza. Notably, pretreatment with gilteritinib did not diminish the cytotoxic effects of chemotherapy, as reported with other FLT3 inhibitors [24, 25]. The cytotoxic effects of chemotherapy were maintained despite decreases in S1 and G_2_/M phase cells and increase in G_1_ phase cells in the gilteritinib treated group. Substantial inhibition of tumor growth was observed in xenografted mice treated with gilteritinib by Day 21, with tumor regression observed following treatment with gilteritinib plus AraC and DNR or gilteritinib plus AraC and IDR. It is difficult to determine whether the efficacy of combination therapy is additive or synergistic; however, the possibility of a synergistic effect remains as gilteritinib in combination with Aza resulted in a significant decrease in tumor volume versus treatment with gilteritinib alone. Treatment with Aza alone had little effect on tumor volume. Also complete regression of the tumor was observed with triple combination therapy but no regression was observed with either gilteritinib or chemotherapy alone. The possibility of drug interaction as a potential mechanism for the effects of combination therapy is unlikely, since there was no discernable difference in the PK profiles of these agents when administered alone or in combination. Conversely, decreased expression of anti-apoptotic proteins MCL-1, survivin, and BCL2L10 by gilteritinib may be an important contributing factor to the efficacy of combination therapy. The decreased expression of MCL-1 following treatment with gilteritinib may have enhanced the sensitivity of MV4-11 cells to AraC and DNR. Notably, the FLT3 inhibitor, Gö6976, induced proteasome-mediated degradation of MCL-1 via a proteasome-mediated mechanism and inhibited survivin expression via STAT 3/5 suppression in MV4-11 and MOLM13 cells [26]. In addition to STAT 3/5 suppression, Gö6976 also inhibited p38MAPK, ERK 1/2, and AKT [26]. Similar suppression of STAT, ERK, and AKT signaling was observed in MV4-11 cells treated with gilteritinib [19]. Evidence from multiple preclinical reports indicates that the downregulation of survivin may enhance sensitivity to chemotherapy in various cancer types [27–29]. Furthermore, downregulation of BCL2L10 expression has been shown to increase sensitivity of myeloid cells to Aza [12]. Findings from our study suggest that gilteritinib-induced downregulation of MCL-1, survivin, or BCL2L10 expression may increase sensitivity to AraC, DNR, and IDR. It is reported that expression of anti-apoptotic proteins may sensitize tumor cells to chemotherapy agents such as etoposide and fludarabine, which are other drugs used to treat AML [30, 31]. It is therefore possible, although not proven, that gilteritinib may also have more potent efficacy when combined with etoposide and fludarabine. AXL has been implicated in chemotherapy resistance as well as resistance to FLT3 inhibitors [9, 10]. Thus, inhibition of AXL by gilteritinib suggests a potential advantage of gilteritinib therapy over treatment with other FLT3 inhibitors. Upregulation of the FLT3 ligand following chemotherapy is another mechanism that contributes to both chemotherapy and FLT3 inhibitor resistance in AML, especially in tumor cells that co-express *FLT3-ITD* and *FLT3* wild-type alleles [32, 33]. Findings from the current study suggest that despite potential increases in the FLT3 ligand induced by chemotherapy, gilteritinib combined with chemotherapy was effective compared with either chemotherapy or gilteritinib alone in both MV4-11 cells that expressed *FLT3-ITD* mutations as well as in MOLM13 cells that expressed *FLT3-ITD* and wild-type *FLT3*. These results suggest that gilteritinib may be potentially efficacious in the presence of the FLT3 ligand, a phenomenon that could be investigated in future studies. As a type I FLT3 inhibitor, gilteritinib inhibits FLT3 in its active form [34]. We have previously reported that gilteritinib inhibited cell growth and suppressed signaling by STAT, ERK, and AKT in Ba/F3 cells that expressed *FLT3-TKD* D835 mutations or *FLT3-TKD* D835 and *FLT3-ITD* mutations [19]. Based on these observations, it is likely that gilteritinib in combination with chemotherapy may be efficacious in tumors expressing *FLT3-TKD* D835 mutations.

The antitumor effects of gilteritinib described in our study corroborate those reported by Mori and colleagues [19]. The addition of AraC/DNR, AraC/IDR, or Aza potentiated the antitumor effects of gilteritinib, suggesting an increased sensitivity to antitumor activity following gilteritinib administration. The potential benefit of combining gilteritinib with chemotherapy in patients with AML is being explored. A phase 1 study (NCT02236013) of gilteritinib plus 7+3 AraC/IDR induction and high-dose AraC consolidation therapy in newly diagnosed AML patients [35], and a phase 2/3 study (NCT02752035) of gilteritinib plus Aza in newly diagnosed *FLT3* mutation-positive AML patients have been initiated [36].

## MATERIALS AND METHODS

### Compounds and cell lines

Gilteritinib (ASP2215), a small molecule tyrosine kinase inhibitor, was synthesized by Astellas Pharma, Inc. (Tokyo, Japan). Gilteritinib was dissolved in dimethyl sulfoxide (DMSO) or was suspended in 0.5% for *in vitro* and *in vivo* experiments, respectively. Cytarabine (Cylocide^®^ injection 60 mg, Nippon Shinyaku Co., Ltd., Kyoto, Japan) was diluted with saline prior to administration. Daunorubicin hydrochloride (Daunomycin^®^ 20 mg, Lot No. 1014, Meiji Seika Pharma Co., Ltd., Tokyo, Japan) was dissolved in saline on the first day and further diluted with saline prior to administration. Idarubicin hydrochloride (Idamycin^®^ 5 mg, Pfizer Inc., New York, NY, USA) was dissolved in distilled water and diluted with saline prior to administration. Azacitidine (5-Azacytidine, A2033, Tokyo Chemical Industry Co. Ltd., Tokyo, Japan) was dissolved in saline prior to administration. Human AML-derived MV4-11 cells (American Type Culture Collection, Manassas, VA, USA) that endogenously expressed *FLT3-ITD* mutations were cultured in Iscove's Modified Dulbecco's Medium (IMDM) supplemented with 10% heat-inactivated fetal bovine serum (FBS) at 37°C in 5% CO_2_. Human AML-derived MOLM-13 cells that endogenously expressed *FLT3-ITD* mutations (German Collection of Microorganisms and Cell Cultures, Braunschweig, Germany) were cultured in Roswell Park Memorial Institute-1640 (RPMI-1640) medium with 10% heat-inactivated FBS at 37°C in 5% CO_2_ [37].

### Cell cycle analysis

MV4-11 cells were seeded in 12-well plates (AGC Techno Glass Co. Ltd., Shizuoka, Japan) at a concentration of 2 × 10^5^ cells/well and cultured overnight. The cells were treated with gilteritinib concentrations of 1, 3, 10, and 30 nM or vehicle (0 nM), and incubated for 24 hours.

The cells were subsequently harvested and fixed in ice-cold 70% ethanol and maintained at 4°C. Following fixation, the cells were washed with phosphate-buffered saline (PBS) and were resuspended in Guava^®^ Cell Cycle Reagent (Merck Millipore Corporation, Darmstadt, Germany). Cell cycle distribution was measured using a Guava^®^ PCA microcytometer (Merck Millipore Corporation), and was analyzed in 5000 cells per sample using CytoSoft™ software (Merck Millipore Corporation). The mean percentages of cells in sub-G1, G1, S, and G2/M phases were derived from four independent assays.

### Annexin-V staining

MV4-11 cells were seeded in 12-well plates at a concentration of 2 × 10^5^ cells/well and cultured overnight. MV4-11 cells were treated with gilteritinib (1, 3, 10, and 30 nM), AraC (1000 nM), DNR (6 nM), IDR (1 nM), Aza (1000 nM), or vehicle (control; 0 nM) and incubated for 48 hours. For experiments involving combination therapy, MV4-11 cells were treated with gilteritinib (1, 3, or 10 nM) in combination with AraC, DNR, IDR, or Aza for 48 hours. For sequential combination treatment experiments, MV4-11 cells were treated with AraC or gilteritinib for 24 hours and further incubated for 48 hours after the addition of gilteritinib or AraC. MOLM-13 cells were seeded in 12-well plates at a concentration of 1 × 10^5^ cells/well and cultured overnight. For experiments where cells were treated with gilteritinib alone, MOLM-13 cells were treated with gilteritinib at concentrations of 0 to 100 nM for 48 hours. For experiments involving combination treatment, the cells were treated with vehicle (0 nM), gilteritinib (30 nM), IDR (3 nM), AraC (100 nM), Aza (1000 nM), gilteritinib plus IDR, gilteritinib plus AraC, or gilteritinib plus Aza (1000 nM) for 48 hours.

Cells were harvested and incubated with Guava^®^ Nexin Reagent (Merck Millipore Corporation, Billerica, MA). Annexin-V-positive cells were assessed using a Guava^®^ PCA microcytometer (Merck Millipore Corporation); the percentage of annexin-V-positive cells (2000 cells per sample) was analyzed using CytoSoft™ software (Merck Millipore Corporation). Mean percentages of annexin-V-positive cells were derived from three independent experiments.

### Western blot analysis

MV4-11 cells were seeded at a concentration of 6 × 10^6^ cells/15 cm dish and were cultured 1 day prior to treatment with control (DMSO), gilteritinib (10 nM), AraC (1000 nM), DNR (6 nM), IDR (1 nM and 2 nM), or Aza (1000 nM) alone, or with gilteritinib in combination with these agents, for 24 hours. MOLM-13 cells were seeded at a concentration of 1.5 × 10^6^ cells/10 cm dish and were cultured 1 day prior to treatment with control (DMSO), gilteritinib (30 nM), AraC (100 nM), IDR (3 nM), or Aza (1000 nM) alone, or gilteritinib in combination with these agents, for 24 hours. Cell lysates were prepared in radioimmunoprecipitation assay buffer (Thermo Fisher Scientific Inc., Rockford, IL) supplemented with phosphatase (Thermo Fisher Scientific Inc.) and protease inhibitors (Merck, Billerica, MA), electrophoresed on 4%–15% gels, and subsequently transferred to polyvinylidene difluoride membranes. The membranes were blocked using Blocking One (Nacalai Tesque Inc., Kyoto, Japan) for 1 hour and then incubated overnight with primary antibodies to MCL-1, BCL210, cPARP (all from Cell Signaling Technology, Danvers, MA, USA), survivin (R&D Systems, Inc., Minneapolis, MN), and α-actin (MilliporeSigma, Burlington, MA). The membranes were washed with TBS Tween-20 (TBS-T) buffer and incubated with appropriate horseradish peroxidase-linked secondary antibodies (Cell Signaling Technology Japan K.K.) for 1 hour at room temperature. After washing the membranes in TBS-T buffer, enhanced chemiluminescence Western Blotting Detection Reagent ECL-prime (GE Healthcare, Fairfield, CT) was applied. Signals were detected with a charge-coupled device camera (ImageQuant LAS4000; GE Healthcare); signal intensity was quantified using ImageQuant™ TL software (GE Healthcare).

### Mouse xenograft models and treatment administration

Four or five-week-old male nude mice (CanN.Cg-Foxn1nu/Crl/Crlj[nu/nu]; Charles River Laboratories Japan, Inc., Kanagawa, Japan) were maintained under specific pathogen-free conditions and received a standard diet and water supply throughout the study. All animal experimental procedures were performed in accordance with the Animal Ethics Committee of Astellas Pharma, Inc. For *in vivo* experiments, suspensions of MV4-11 and MOLM-13 cells in PBS at a concentration of 1 × 10^8^ cells/mL were mixed with an equivalent volume of Matrigel^®^ Basement Membrane Matrix, resulting in a final cell concentration of 5 × 10^7^ cells/mL. The cells were subcutaneously injected into the flanks of mice at a concentration of 5 × 10^6^ cells/0.1 mL/mouse. After tumor establishment, mice were divided into groups with similar mean tumor volume across groups.

Dosing volumes were 10 mL/kg for oral and intraperitoneal (IP) administration, and 5 or 10 mL/kg for intravenous (IV) administration. Treatment was administered once daily on designated days. The first day of treatment was designated as Day 0 and observation continued until Day 20 or 21 for mice xenografted with MV4-11 cells and Day 16 for mice xenografted with MOLM-13 cells. In mice xenografted with MV4-11 cells for experiments involving triple combination treatment, the control group received oral administration of vehicle (0.5% methylcellulose, Days 0–20), IP administration of vehicle (saline, Days 0–4), and IV administration of vehicle (saline, Days 0–2). The gilteritinib-only group received 3 mg/day gilteritinib (PO, Days 0–20) and vehicle (IP, Days 0–4 and IV, Days 0–2).

The AraC/DNR group received oral vehicle (0.5% methylcellulose, Days 0–20), IP AraC (50 mg/kg/day, Days 0–4), and IV DNR (2 mg/kg/day, Days 0–2). The AraC/IDR group received vehicle (Days 0–20), IP AraC (50 mg/kg/day, Days 0–4), and IV IDR (0.5 mg/kg/day, Days 0–2). The triple combination groups received oral gilteritinib (3 mg/kg/day, Days 0–20) plus IP AraC (50 mg/kg/day, Days 0–4) plus either IV DNR (2 mg/kg/day, Days 0–2) or IV IDR (0.5 mg/kg/day, Days 0–2). For experiments involving Aza, the control group received oral vehicle (0.5% methylcellulose, Days 0–20) and IV vehicle (saline, Days 0–4). The Aza group received oral vehicle (0.5% methylcellulose, Days 0–20) and IV Aza (3 mg/kg/day, Days 0–4). The gilteritinib group received oral gilteritinib (3 mg/kg/day, Days 0–20) and IV vehicle (saline, Days 0–4). Mice in the combination treatment group received oral gilteritinib (3 mg/kg/day, Days 0–20) and IV Aza (3 mg/kg/day, Days 0–4). For experiments involving triple combination sequential treatment, xenografted mice in the concomitant combination group received gilteritinib orally (3 mg/kg/day, Days 0–21), IP AraC (50 mg/kg/day) plus IV DNR (2 mg/kg/day, Days 0–2). Xenografted mice in the sequential combination group received gilteritinib (3 mg/kg/day, Days 0–21) followed by AraC (50 mg/kg/day, Days 7–11) plus DNR (2 mg/kg/day, Days 7–9). For mice that received chemotherapy only, administration schedules for each agent were the same as those described in the triple combination groups.

Mice xenografted with MOLM-13 cells received gilteritinib (30 mg/kg/day, Days 0–15 or 16); IDR, AraC, and Aza were administered using the same doses and schedules as those described for mice xenografted with MV4-11 cells.

### Tumor evaluation

Mice with established tumors were selected and divided into groups on Day −1 or Day 0, so that the mean tumor volumes were similar across groups. Body weights and tumor volumes assessed on Day −1 were designated as Day 0 values. Body weight and tumor diameter were measured for each experimental group; antitumor activity was expressed as the percent inhibition of tumor growth and the percent of tumor regression (further details are outlined in the Supplementary Materials). When percent inhibition of tumor growth exceeded 100%, percent tumor regression was calculated. Complete regression was defined as no palpable or observable tumor.

### Pharmacokinetic assays

Pharmacokinetic parameters were assessed in plasma and tumor fractions obtained from nude mice xenografted with MV4-11 cells following treatment with gilteritinib, AraC, IDR, and Aza alone or in combination (combination treatment groups are described in the Supplementary Materials).

Blood samples were collected at 5 minutes and up to 24 hours post dose from the inferior vena cava. Plasma samples were prepared by centrifugation (n=3 per group; one sample in the combination group was excluded due to improper handling). Tumor samples were collected at 5 minutes and up to 24 hours post dose and tumor weights were measured; plasma and tumor drug concentrations were measured using high-performance liquid chromatography-tandem mass spectrometry. These assays were conducted by Astellas Research Technologies Co., Ltd. under the guidance of Analysis & Pharmacokinetics Research Laboratories, Astellas Pharma, Inc., or by Nemoto Science Co., Ltd. (Ibaraki, Japan). Standard PK parameters were calculated at Analysis & Pharmacokinetics Research Laboratories, Astellas Pharma, Inc.

### Statistical analysis

Cell cycle data analyses and statistical analyses of annexin-V-positive cells were performed using GraphPad Prism (GraphPad Software, San Diego, CA, USA). Mean percentages of cells at cell cycle phases (sub-G1, G1, S, and G2/M), and the mean percentages of annexin-V-positive cells, were calculated and reported as mean ± standard error of the mean (SEM). The Dunnett's multiple comparison test was used for cell cycle analyses (using within subject error) and annexin V-positive cells to compare mean percentages across treatment groups with statistical significance established at *P*<.05. For statistical analysis of annexin-V-positive cells following combination treatment, it was considered significant if combination treatment was more potent than treatment with either agent alone (*P*<0.05; Student's *t*-test). Mean (±SEM) drug concentrations in plasma and tumor fractions of xenografted mice were calculated using n=3 per post-dose time point in all experiments except for the plasma fraction of the 5-minute post-dose time point after treatment with gilteritinib plus Aza, which was based on n=2. Statistical analyses for tumor assessment were performed using SAS software (SAS Institute, Cary, NC, USA). Tumor volume, body weight, and tumor weight were expressed as means (±SEM). The Student's *t*-test was used to compare tumor volume on Day 21 or Day 16 in mice receiving triple combination therapy (ie, gilteritinib plus AraC/DNR, or gilteritinib plus AraC/IDR) with tumor volume in mice receiving gilteritinib alone or AraC plus DNR or AraC plus IDR, as well as tumor volume between mice receiving gilteritinib plus Aza versus those receiving gilteritinib alone or Aza alone. Statistical significance was established at *P*<0.05.

## SUPPLEMENTARY MATERIALS


