# Supplementary material for: Evaluation of gilteritinib in combination with chemotherapy in preclinical models of FLT3-ITD+ acute myeloid leukemia
Source: Oncotarget. 2019 Apr 2;10(26):2530–45. doi: 10.18632/oncotarget.26811 (PMC6493465; doi:10.18632/oncotarget.26811)
Supplement: Supplementary file 1 [file oncotarget-10-2530-s001.pdf]

Evaluation of gilteritinib in combination with chemotherapy in preclinical models of *FLT3-ITD*+ acute myeloid leukemia

SUPPLEMENTARY MATERIALS

Tumor evaluation

Body weight and tumor diameter were measured in xenografted nude mice xenografted with MV4-11 cells on the indicated days for each experimental group: 1. Gilteritinib plus AraC/DNR: Days −1, 3, 6, 9, 14, 17, and 21; 2. Gilteritinib plus AraC/IDR: Days 0, 3, 7, 10, 14, 17, and 21; 3. Gilteritinib plus Aza: Days −1, 2, 5, 8, 12, 15, 19, and 21. Body weight and tumor assessments obtained on Day −1 were designated as Day 0 (baseline) measurements. Tumor diameter was measured using a caliper and tumor volume was determined by calculating the volume of an ellipsoid (ie, $Length \times Width^2 \times 0.5$). The percent inhibition of tumor growth was calculated using the formula:

100 × (1 – [mean {(tumor volume of each mouse on the last day of observation) – (tumor volume of each mouse on Day 0]} of each group] / [mean {(tumor volume of each mouse on the last day of observation) – (tumor volume of each mouse on Day 0)} of the control group])

The percent tumor regression was calculated using the formula:

100 × (1 – [mean tumor volume of each group on the last day of observation] / [mean tumor volume of each group on Day 0])

Treatments for pharmacokinetic assays

For pharmacokinetic (PK) assays involving gilteritinib plus AraC and IDR, nude mice xenografted with MV4-11 cells received one of the following combination treatments: 1. Oral gilteritinib (3 mg/kg in 0.5% methylcellulose); 2. Oral methylcellulose (0.5%) in combination with IV IDR (0.5 mg/kg) and IP AraC (50 mg/kg); 3. Oral gilteritinib (3 mg/kg) plus IV IDR (0.5 mg/kg) and IP AraC (50 mg/kg). For combination PK assays involving gilteritinib plus Aza, nude mice xenografted with MV4-11 received one of the following:

1. Oral gilteritinib (3 mg/kg) plus IV saline; 2. Oral methylcellulose (0.5%) plus IV Aza (3 mg/kg); 3. Oral gilteritinib (3 mg/kg) plus IV Aza (3 mg/kg).
